# The embodied digital divide: how sensorimotor experience shapes touchscreen typing performance and strategy

**DOI:** 10.1007/s00221-025-07220-7

**Published:** 2026-02-03

**Authors:** Laila Craighero, Elisa Straulino, Alba Liso, Lorenzo Viviani, Davide Conte, Alberto Morelli, Leonardo Bocchi, Luisa Sartori

**Affiliations:** 1 Department of Neuroscience and Rehabilitation, University of Ferrara, Via Fossato di Mortara 19, 44121 Ferrara, Italy; 2 Department of General Psychology, University of Padova, Via Venezia 8, 35131 Padua, Italy; 3 Department of Medical Sciences, University of Ferrara, Via Fossato di Mortara 64/B, 44121 Ferrara, Italy; 4 Department of Information Engineering, University of Florence, Via S.Marta, 3, 50139 Florence, Italy

**Keywords:** Movement Vigor, Embodied Cognition, Sensorimotor Representations, Smartphone Interaction, Digital Natives, Digital Immigrants

## Abstract

This study investigated how divergent sensorimotor experiences interact with task intention to influence touchscreen typing performance, kinematics, and strategy in smartphone users. Building on principles of motor learning and embodied cognition, we compared two generational cohorts: individuals whose sensorimotor development was primarily in the physical world, and those whose development occurred concurrently in both physical and digital environments. Participants typed the same sentences in two tasks: WhatsApp Chat (content generation) and Google Search (content consumption). 3D motion capture measured thumb kinematics; video analysis assessed behavioral metrics including typing time, predictive text use, and errors. Behavioral metrics revealed that the group with early digital exposure demonstrated faster overall typing times. The group with later digital adoption showed significantly greater use of predictive text, especially in WhatsApp, while typing errors did not differ. Kinematic analysis showed the early-digital-exposure group exhibited a higher-vigor motor execution profile and later peak deceleration and velocity, indicative of more ballistic, pre-planned movements. Crucially, Movement Time also exhibited a complex interaction, highlighting context-dependent modulation of motor efficiency. Thumb usage patterns did not show major group or task differences. These findings demonstrate that an individual's sensorimotor history contributes to shaping fine-grained sensorimotor skills. Those with early digital exposure display superior motor efficiency and vigor. In contrast, individuals with later digital adoption, though less kinematically efficient, exhibited remarkable strategic flexibility, adapting motor control and leveraging interface features based on task demands. This underscores how divergent sensorimotor histories contribute to distinct expressions of digital fluency and adaptive strategies in the digital age.

## Introduction

In recent decades, digital technologies—most notably smartphones equipped with touchscreen interfaces—have become pervasive, fundamentally reshaping how people access information, communicate, and interact with their environment. This shift has brought about a growing reliance on a set of sensorimotor skills that differ markedly from those used when manipulating physical objects or operating traditional input devices such as keyboards and mice. Everyday actions like typing, swiping, and tapping on a touchscreen require finely tuned, coordinated movements, often executed rapidly and with limited visual monitoring of the fingers.

Compared with conventional physical keyboards, touchscreen typing introduces a distinct set of sensorimotor challenges. The reduced key size, absence of haptic feedback, and transition from ten-finger touch typing to predominantly one- or two-thumb input impose new demands on motor control and necessitate specific adaptive strategies (Hoggan et al. 2008; Xiong and Muraki 2014; Viviani et al. 2025). Among these, thumb typing has emerged as a unique mode of text entry, characterized by its ergonomic constraints and by a notable shift in the thumb’s functional role—from providing stabilization to acting as the primary effector in text production (Xiong and Muraki 2014; Ramati 2023).

The acquisition and refinement of motor skills are shaped by well-established principles of motor learning. As individuals accumulate experience with a given motor task, performance generally improves, exhibiting greater efficiency, shorter reaction times, and reduced movement variability (Davids et al. 2003). Although motor learning has been extensively investigated in domains such as physical skill acquisition and tool use, comparatively little is known about the sensorimotor adaptations and degrees of automaticity that emerge during prolonged interaction with digital touchscreens—devices now embedded in everyday life.

Optimal control frameworks propose that motor vigor reflects a trade-off between the incentive to achieve a goal rapidly and the energetic costs associated with executing faster movements (Shadmehr et al. 2010, 2019; Summerside et al. 2018; Thura et al. 2025). Under the assumption that the brain selects motor commands to maximize reward while minimizing effort, the key determinant of movement execution becomes the subjective value of the reward. When reward value is high, movements display increased peak velocity and sharper acceleration–deceleration profiles (i.e., greater movement vigor; Mazzoni et al. 2007). Elevated vigor, therefore, constitutes a robust behavioral marker of heightened motivation.

Furthermore, the embodied cognition framework posits that cognitive processes are deeply rooted in the interactions of the body with its environment (Wilson 2002; Gallese and Lakoff 2005; Anderson et al. 2012). This perspective suggests that our sensorimotor experiences shape not only how we perform actions but also how we perceive and understand the world. A key notion within this framework is the concept of affordances—sensory characteristics of objects or environments that intuitively suggest possible actions and functions (Gibson 1977; Norman 2016). These affordances are not fixed properties of the environment but are relational, depending on the individual's capabilities and goals. In the context of digital interfaces, 'digital affordances' are the cues through which the user interface communicates its interactivity (Urbano et al. 2020). Critically, the nature and interpretation of digital affordances have evolved over time, often moving from skeuomorphic designs that mimicked physical objects (e.g., trash bin, envelope) to more abstract flat designs (e.g., arrow), requiring the acquisition of new knowledge and sensorimotor skills for effective interaction.

Applying the embodied cognition perspective to the digital age, it is plausible that the extensive and often qualitatively different sensorimotor experiences of interacting with digital devices have reshaped human cognition and its neural substrate. While traditional sensorimotor experiences in the physical world have been relatively stable across generations, the rapid evolution and widespread adoption of digital technology have introduced entirely new patterns of interaction. In this context, for individuals often categorized as Digital Immigrants (DI, those who adopted digital technology later), sensorimotor experiences and the associated brain adaptations primarily occurred through interaction with the physical environment. Their understanding of "affordances" and motor control was largely forged in a world of tangible objects and physical manipulations (Craighero 2014). In contrast, Digital Natives (DN, those born into the digital world) have grown up with pervasive digital technology, developing sensorimotor skills and interacting with digital affordances from an early age. Their sensorimotor experiences have unfolded in parallel in both the physical and digital worlds, potentially leading to distinct sensorimotor representations and cognitive strategies for interacting with each domain. This generational difference in sensorimotor "training" raises important questions about how these distinct experiences might influence the efficiency and kinematic profiles of digital actions and the neural substrates that support them.

Despite the centrality of sensorimotor interaction in the digital age, neuroscientific and behavioural research has only recently begun to explore its impact on the brain and behaviour. Most studies have focused on short-term adaptations or general usage patterns (e.g., Craighero & Marini 2021; Ghosh et al. 2017). However, a critical gap exists in understanding the fine-grained motor control employed during specific, common digital tasks and how this might be modulated by individual factors such as digital experience or generational differences. It is commonly observed that individuals belonging to different generational cohorts may interact with technology in qualitatively different ways. While the strict boundary based on birth year (Prensky 2001) is increasingly blurred due to widespread technology adoption (Toledo 2007), and while simple differences in habitual use and proficiency may no longer be universally granted, distinct sensorimotor interaction histories undoubtedly persist.

Another factor that systematically modulates the execution of the gesture is the purpose of the action, that is, the goal with which it is performed. Decades of research have shown that the kinematic profile of an action is not solely determined by the effector (e.g., hand or foot), the target size or the immediate motor goal (e.g., grasping an object), but is also profoundly shaped by the ultimate intention or purpose behind the action (Becchio et al. 2010; Sartori et al. 2011). For instance, the kinematics of a reach-to-grasp movement differ subtly yet reliably depending on whether the object is being grasped for immediate use or for placing it elsewhere, with these differences observable even in the initial phases of the movement (Sartori et al. 2011). These goal-dependent modulations in movement kinematics can be perceived by observers to infer human intent (Soriano et al. 2018). Such findings highlight that motor planning and execution are tightly coupled with the cognitive representation of the action's overall goal.

Extending this principle to the digital realm, it is plausible that common touchscreen actions, such as typing, may also be tailored to the specific digital intention (Craighero et al. 2023). For instance, composing a text message to a friend differs in context and purpose from searching the same information online, even if both involve typing the same sentence on a virtual keyboard. This task distinction may also relate to cognitive representations of space. Research in physical interaction has long differentiated between "near" or "peripersonal" space (within reach) and "far" or "extrapersonal" space (beyond reach), showing that actions directed towards these different spatial domains recruit distinct neural networks. Interestingly, recent findings suggest a similar distinction might exist in the digital environment, where tasks involving content generation or communication (e.g., chatting) are cognitively associated with "near" space, while tasks involving content consumption or information retrieval (e.g., searching) are linked to "far" space (Craighero & Marini 2021).

Understanding if these differing digital intentions are reflected in measurable kinematic parameters could provide valuable insights into the sensorimotor "syntax" of digital interaction and serve as an objective index of digital capacity, reflecting how users adapt their motor behavior to different digital goals.

In the present study, we employed 3D motion capture to investigate the kinematic characteristics of thumb movements during two common smartphone-based typing tasks: chatting via WhatsApp (representing content generation/communication) and searching via Google (representing content consumption/information retrieval). We analyzed temporal, spatial, and speed parameters of movements. Furthermore, we conducted a comprehensive analysis of video recordings of the participants' typing behavior. This video analysis allowed us to identify typing errors, to quantify the use of text suggestions, and to map the spatial distribution of key presses across the keyboard for both the right and left thumb. We collected these kinematic and behavioral data for participants categorized as either Digital Natives or Digital Immigrants.

By comparing the kinematic profiles and behavioral metrics across tasks and generational cohorts, we aimed to characterize the motor efficiency and strategies employed during different digital interactions and explore the influence of digital experience.

## Materials and methods

### Participants

A total of 39 participants (21 males, 18 females) were recruited for this study through university announcements and provided written informed consent prior to participation. Inclusion criteria were right-handedness assessed by the Edinburgh Handedness Inventory (Oldfield 1971), typing with two thumbs, self-reported normal or corrected-to-normal vision, and no history of neurological or motor disorders. Five participants were excluded due to left-handedness, and one participant was excluded due to an atypical typing style (using the index and middle fingers of the right hand instead of the thumb). The final sample included 33 right-handed participants (17 males, 16 females). Participants were assigned to one of two groups, serving as proxies for distinct digital sensorimotor histories, based on their year of birth. This categorization followed the common distinction proposed by Prensky (2001): the Digital Immigrants (DI) group, representing individuals whose primary sensorimotor development largely preceded widespread digital adoption, was defined as individuals born before 1980; and Digital Natives (DN) group, representing individuals who grew up immersed in pervasive digital technology, was defined as individuals born in 1980 or later. The DI group consisted of 10 participants (mean age = 58.3, range = 49–70 years), and the DN group consisted of 23 participants (mean age = 23.7, range = 20–29 years). All participants were naive to the purpose of the study.

Participants were also screened for their familiarity with the specific apps used in the study. We verified that Google was the default search engine on all participants' devices. Additionally, participants rated their WhatsApp usage frequency on a 3-point Likert scale (Sometimes, Frequently, Always).

The study was conducted in accordance with the ethical principles outlined in the Declaration of Helsinki. The experimental protocol was approved by the Ethical Committee of University of Padova (protocol Nr. 141-a).

### Subsamples for specific analyses

Due to specific data quality requirements and analytical approaches, the participant samples varied slightly across the different analyses conducted:

Kinematic Analysis: For the detailed kinematic analysis of consecutive key presses, stringent data quality criteria (e.g., sufficient number of comparable consecutive thumb movements) led to the inclusion of a subsample of 13 participants. This subsample consisted of six DI (3 females, 3 males; mean age = 58.33 years, SD = 4.76, age range = 50–63) and seven DN (2 females, 5 males; mean age = 24.43 years, SD = 2.07, age range = 22–27). For the non-dominant Left Hand, strictly consecutive movements were rarer in the older group; consequently, only three of the six DI participants included in the right-hand analysis provided sufficient data for the left hand, while the DN subsample remained unchanged (n = 7). Importantly, both groups showed a nearly identical high-usage profile for WhatsApp: 5 DN and 5 DI participants reported using the app 'Always,' while the remaining participants (2 DN, 1 DI) reported using it 'Frequently.'

Behavioral Metrics (Typing Time, Predictive Text Use, Typing Errors): A subsample of 21 participants was selected to ensure balanced group sizes. As a strategy to achieve this balance, we decided to include data from only the DN recruited during the first two days of data collection (n = 11), alongside all 10 DI. However, one DI participant was excluded due to incomplete data. The resulting subsample consisted of 9 DI (4 females, 5 males; mean age = 58.2 years, range = 49–70) and 11 DN (3 females, 8 males; mean age = 23.1 years, range = 20–26). Regarding task familiarity, both groups exhibited comparable high-usage patterns for WhatsApp: 7 DN and 6 DI participants reported using the application 'Always,' while the remaining participants (4 DN and 3 DI) reported using it 'Frequently.'

Thumb Usage Patterns: The analysis of thumb usage patterns required specific video analysis criteria (e.g., clear visibility of thumb contact for each key press, consistent two-thumb typing throughout). This led to a subsample of 20 participants for this specific analysis. This subsample included 10 DI (4 females, 6 males; mean age = 58.3 years, range = 49–70) and 10 DN (4 females, 6 males; mean age = 23.45 years, range = 20–26), selected to ensure an equal representation of both groups. Participants were excluded if they did not perform the task using both thumbs, if the number of errors or use of suggestions prevented the collection of analyzable letters, or if video analysis did not allow for a clear determination of thumb usage for specific key presses. Crucially, the two groups showed an identical high-usage profile for WhatsApp: in both the DN and DI cohorts, 7 participants reported using the application 'Always,' and 3 reported using it 'Frequently.'

### Apparatus, stimuli, and procedure

Typing movements were recorded using an optoelectronic three-dimensional motion analysis system (SMART-D; Bioengineering Technology & Systems, BTS) equipped with six infrared cameras (sampling frequency: 70 Hz), which enabled the three-dimensional coordinates (X,Y,Z) of infrared-reflective markers (6 mm diameter) with a hemispherical shape to be estimated with considerable precision at each time frame. Two markers were placed on the distal phalanx (fingertip) of the right and left thumb using adhesive tape. The six cameras were arranged in a semicircle around the participant. Prior to data collection, the image captured by each camera was optimized by adjusting its zoom, focus, threshold and brightness. The system was then calibrated in order to define an absolute spatial reference system. For the static calibration, a three-axes frame of nine markers at known distances from each other was placed in the middle of the table. For the dynamic calibration, a three-marker wand was moved throughout the workspace of interest for 60 s. The spatial resolution of the recording system was 0.3 mm over the field of view. The standard deviation of the reconstruction error was 0.2 mm for the x, y and z axes.

Concurrent with motion capture, video recordings capturing the participants' hands and the smartphone screen from above were acquired using an external video camera placed directly above the participant's hands and the device. This video feed provided a detailed view of the typing process and allowed for frame-by-frame analysis using Kinovea® software (Kinovea; Version 0.8.15; Kinovea open source project http://www.kinovea.org) (Fernández-González et al. 2020). This video analysis was critical for several aspects of data processing: identifying the sequence of key presses and consecutive thumb movements for subsequent kinematic analysis, and extracting behavioral data, including typing time (defined as the duration from the first to the last keystroke for each sentence), the number of predictive text selections, and the number of typing errors and corrections (reversals, omissions, additions). Furthermore, it provided the raw data for the detailed analysis of thumb usage described below.

Participants performed two smartphone-based typing tasks using their own smartphone. The two tasks differed in the participant's digital intention:

- WhatsApp Chat: Participants were instructed to type the sentences during a chat conversation with a contact person.

The cover story and instructions provided to participants for this task were as follows:

“Call your contact person. You will need to ask for their help using words similar to these:

'I need to participate in a game and I need your help. We need to answer a series of questions and then compare our answers with those given by Google. Are you ready to help me?'.

Once your contact is ready, the experimenter will give you one question at a time, and you must ask it to your contact person. Pay attention, because the words must be exactly as communicated!

After each question, continue the discussion naturally until you agree on an answer. Only your contact can go on Google; you must wait for the information to be given to you by the contact person before communicating it to the experimenter."

This task was designed to simulate content generation and communication in a natural conversation flow.

- Google Search: Participants were instructed to type the same sentences directly into the Google search bar.

The instructions were: "Now you will need to search for the answers to the questions on Google and then compare them with those found by your contact person."

This task was designed to simulate content consumption and information retrieval.

The tasks involved typing seven specific sentences selected based on the following criteria: avoidance of accents or apostrophes, avoidance of unusual or potentially error-prone words, avoidance of questions with obvious or easily known answers, and a hypothesised balance in the number of key presses required for the left and right sides of the keyboard. The translation of the Italian sentences used in the tasks is: How long do boiled potatoes need to boil? How long does grandmother's apple pie bake? How long is a passport valid for? How long does an electric car take to charge? How much does a relaxing massage cost? How many years does an elephant live in the savannah? How much flour should be put in gnocchi?

At the beginning of each session (WhatsApp Chat or Google Search), general instructions for that task were provided to the participant.

Prior to each sentence, participants rested their hands comfortably on the table in a spontaneous position. For each of the seven sentences, the following procedure was then repeated: the experimenter verbally read the sentence to the participant; the participant used their right hand to grasp their smartphone, which was placed on the table at a distance of 35 cm from this starting position; the participant then typed the required sentence using the relevant application for the session (WhatsApp or Google Search); after receiving the answer from the chat partner (in the WhatsApp session) or finding the answer via Google search (in the Google session), the participant returned the smartphone to the table in its starting position.

The order of the two tasks (WhatsApp Chat and Google Search) was counterbalanced across participants.

## Data analysis and results

The data used in this study can be found on the Open Science Framework at https://osf.io/wcsp9

### Kinematics data

Given the novelty of detailed kinematic analysis applied specifically to touchscreen typing, we adapted analytical approaches commonly used in the study of reaching movements in the physical world (Jeannerod 1984). Specifically, we focused our kinematic analysis on the movement of the thumb between consecutive key presses performed by the same thumb. This approach was necessary because the kinematic profile of movements between non-consecutive keys within a typed sequence is often influenced by pauses or preparatory movements made while the other thumb is active. To ensure a sufficient number of comparable data points for this analysis, we applied specific criteria for participant and movement selection. We included only data from participants who consistently used the same thumb to type at least two consecutive keys in both the WhatsApp Chat and Google Search sessions. Furthermore, the kinematic analysis was restricted to movements between pairs of consecutive keys that were typed by the same thumb across all selected participants in both sessions.

After data acquisition, raw IR-cameras data were pre-processed using the SMART-D Tracker (BTS SpA) software to reconstruct 3D trajectories of thumbs and phone markers. Subsequently, the SMART-D Analyzer (BTS SpA) software was used to filter and analyse thumbs trajectories with a protocol specifically designed to detect temporal and speed parameters as dependent variables. Markers trajectories were filtered using a fourth-order low-pass Butterworth filter with cut-off frequency @ 10 Hz (Crenna et al. 2021) and their first and second time-derivatives (i.e. velocity and acceleration) were computed by digital differentiation. Time-instants of meaningful gesture events were identified based on velocity and acceleration traces.

Custom MATLAB functions to estimate thumbs-to-phone screen contact events during the typing phase were developed, based on the analysis of thumbs and phone markers trajectories. Phone markers were used to estimate the spatial orientation of the plane containing the phone screen surface during the typing phase. Then, a frame-by-frame value of orthogonal distance of each thumb marker from this plane was computed. Based on the hypothesis that typing events correspond to local minima of the thumb-to-screen distance curve, a user-supervised algorithms of typing events detection was developed and is described in the following: for each specific trial, every single letter composing the sentence was classified as a right- or a left-side typing during the video analysis performed with Kinovea® software, described above. This a-priori information was then used by the algorithm to try an automatic association of each single letter of the sequence with local minima of, respectively, the right or the left thumb-to-screen distance curves. An interactive plot of curves estimated typing events and associated letters allowed the user to check if the letters sequence was reconstructed in the correct order, with the possibility to manually cancel/add events until perfect alignment, with optional support of video recordings inspection.

The present research focuses on paired sentence segments across different conditions. We then analyzed consecutive basic actions to determine whether there are significant differences in the kinematic parameters of typing in either content generation or content consumption (Table 1). Consecutive basic actions are defined as a sequence of fundamental motor actions performed one after another in a continuous manner, where each action is a discrete, minimal unit of movement necessary to achieve a more complex motor task. Specifically, in this study we sought to examine the typing differences between two groups, representing divergent sensorimotor histories (Digital Natives and Digital Immigrants), under two distinct Conditions: interactive content generation on WhatsApp and passive content consumption on Google.

**Table 1:** Number of consecutive basic actions included in the kinematic analysis, separated by Group and Hand. Note: The number of actions included for the right hand differs from that for the left hand, reflecting variations in the availability of comparable consecutive thumb movements for each hand

Group	MP	PO	VO	NO	BO	LL	LI	IN	UO	ON	NN	IL	UN	HI	NI	IV	NT	TT	IC	IO	BI	OG	GN	GG
Right Hand Basic Actions																								
DI	6	6	0	3	0	4	5	10	0	1	4	2	6	2	3	1	2	2	0	1	0	0	0	1
DN	14	18	2	5	3	6	9	10	4	4	5	7	12	2	5	5	9	4	4	4	3	1	1	4

Group	ER	DE	RE	ES	SE	TE	CE	AS	SA	CA	AC	CC	AR	EG	SS	RT	TT	TR	GG	EG				
Left Hand Basic Actions																								
DI	4	2	1	1	1	7	0	3	2	2	1	3	2	1	3	0	2	1	1	1				
DN	9	4	8	5	1	12	4	12	11	1	0	3	3	1	13	5	4	1	4	1				

For each selected consecutive key press, the following metrics were computed:

- Movement Time (MT): the time between movement onset (i.e., the moment the thumb was lifted from a key) and movement offset (i.e., the moment it contacted the next key) during smartphone typing.
- Maximum Velocity (MV): the maximum value of thumb 3-D velocity (mm/s).
- Maximum Deceleration (MDec): the absolute value of the maximum rate at which the thumb slows down (mm/sec2).
- Time to Maximum Velocity% (TMV%): the instant at which the thumb velocity is maximum from the start of the movement (normalized on movement time).
- Time to Maximum Deceleration% (TMDec%): the instant at which the thumb deceleration is maximum from the start of the movement (normalized on movement time).

Data were analysed using JASP version 0.19.3 (JASP Team, 2025) statistical software. Moreover, we calculated the nine deciles for each individual's variable and excluded all data that fell outside the range between the 1st and 9th deciles. This approach allowed us to obtain more consistent data for each participant by excluding extreme values and outliers.

For each kinematic metric, a mixed analysis of variance (ANOVA) was conducted on the mean values calculated across all valid basic actions for each participant within each condition, with Hand (Right, Left), Task (WhatsApp Chat, Google Search) as the within-subjects factors and Group (Digital Immigrants, Digital Natives) as the between-subjects factor. Where significant effects were found, Bonferroni-corrected post-hoc comparisons were performed. To explore in detail how group differences manifested specifically for each hand, separate mixed-design ANOVAs were subsequently conducted for the right and left hands.

Alpha level was set at *p* < 0.05. We then calculated the post hoc power using G*Power 3.1 (Erdfelder et al., 1996) and we found that the power was 0.94 (Effect size f = 0.42, alpha = 0.05, total sample size = 13).

All statistical values for the global mixed-design ANOVAs and the separate mixed-design ANOVAs by hand are reported in Table 2.

**Table 2:** Summary of Mixed-Design ANOVA Results for Kinematic Parameters, including Main Effects and Interactions of Group, Task, and Hand. Both global ANOVA results and separate ANOVA results for each hand are presented. Bolded p-values indicate statistical significance (p < .05)

Kinematic Parameter	Main Effect Hand	Main Effect Task	Main Effect Group	Interaction Hand x Task	Interaction Hand x Group	Interaction Task x Group	Three-way interaction Hand x Task x Group
MT	*F*_*(1,8)*_ = 0.13 *p* = 0.73 *η*^*2*^_*p*_ = 0.02	*F*_*(1,8)*_ = 0.83 *p* = 0.39 *η*^*2*^_*p*_ = 0.09	***F***_***(1,8)***_** = 18.06** ***p* = 0.003** ***η***^***2***^_***p*=**_**0.69**	*F*_*(1,8)*_ = 1.33 *p* = 0.28 *η*^*2*^_*p*_ = 0.14	*F*_*(1,8)*_ = 0.46 *p* = 0.51 *η*^*2*^_*p*_ = 0.05	*F*_*(1,8)*_ = 0.08 *p* = 0.78 *η*^*2*^_*p*_ = 0.01	***F***_***(1,8)***_** = 5.83** ***p* = 0.04** ***η***^***2***^_***p***_** = 0.42**
	*Right hand*	*F*_*(1,11)*_ = 0.06 *p* = 0.81 *η*^*2*^_*p*_ = 0.00	***F***_***(1,11)***_** = 31.82** ***p* < 0.001** ***η***^***2***^_***p***_** = 0.74**			*F*_*(1,11)*_ = *0.005* *p* = *0.95* *η*^*2*^_*p*_ = *0.00*	
	*Left hand*	*F*_*(1,8)*_ = *0.007* *p* = *0.94* *η*^*2*^_*p*_ = *0.00*	***F***_***(1,8)***_** = 13.31** ***p* = 0.006** ***η***^***2***^_***p***_** = 0.62**			*F*_*(1,18*_ = *1.31* *p* = *0.29* *η*^*2*^_*p*_ = *0.14*	
MV	***F***_***(1,8)***_** = 20.94** ***p* = 0.002** ***η***^***2***^_***p***_** = 0.72**	*F*_*(1,8)*_ = 0.25 *p* = 0.63 *η*^*2*^_*p*_ = 0.03	*F*_*(1,8)*_ = 1.40 *p* = 0.27 *η*^*2*^_*p*_ = 0.15	*F*_*(1,8)*_ = 0.30 *p* = 0.59 *η*^*2*^_*p*_ = 0.04	*F*_*(1,8)*_ = 0.83 *p* = 0.39 *η*^*2*^_*p*_ = 0.09	*F*_*(1,8)*_ = 0.31 *p* = 0.59 *η*^*2*^_*p*_ = 0.04	*F*_*(1,8)*_ = 2.56 *p* = 0.15 *η*^*2*^_*p*_ = 0.24
	*Right hand*	*F*_*(1,11)*_ = 1.11 *p* = 0.31 *η*^*2*^_*p*_ = 0.09	***F***_***(1,11)***_** = 6.21** ***p* = 0.03** ***η***^***2***^_***p***_** = 0.36**			*F*_*(1,11)*_ = 0.12 *p* = 0.73 *η*^*2*^_*p*_ = 0.01	
	*Left hand*	*F*_*(1,8)*_ = 0.53 *p* = 0.48 *η*^*2*^_*p*_ = 0.06	*F*_*(1,8)*_ = 0.22 *p* = 0.65 *η*^*2*^_*p*_ = 0.03			*F*_*(1,8)*_ = 1.86 *p* = 0.21 *η*^*2*^_*p*_ = 0.19	
MDec	***F***_***(1,8)***_** = 10.89** ***p* = 0.01** ***η***^***2***^_***p***_** = 0.58**	*F*_*(1,8)*_ = 0.52 *p* = 0.49 *η*^*2*^_*p*_ = 0.06	***F***_***(1,8)***_** = 6.26** ***p* = 0.04** ***η***^***2***^_***p***_** = 0.44**	*F*_*(1,8)*_ = 1.21 *p* = 0.30 *η*^*2*^_*p*_ = 0.13	*F*_*(1,8)*_ = 1.77 *p* = 0.22 *η*^*2*^_*p*_ = 0.18	*F*_*(1,8)*_ = 0.89 *p* = 0.37 *η*^*2*^_*p*_ = 0.10	*F*_*(1,8)*_ = 2.50 *p* = 0.15 *η*^*2*^_*p*_ = 0.24
	*Right hand*	*F*_*(1,11)*_ = 0.02 *p* = 0.89 *η*^*2*^_*p*_ = 0.00	***F***_***(1,11)***_** = 10.89** ***p* = 0.007** ***η***^***2***^_***p***_** = 0.50**			*F*_*(1,11)*_ = *3.50* *p* = 0.09 *η*^*2*^_*p*_ = 0.24	
	*Left hand*	*F*_*(1,8)*_ = 1 *p* = 0.35 *η*^*2*^_*p*_ = 0.11	*F*_*(1,8)*_ = 4.66 *p* = 0.06 *η*^*2*^_*p*_ = 0.37			*F*_*(1,8)*_ = 0.00 *p* = 0.99 *η*^*2*^_*p*_ = 0.00	
TMV%	*F*_*(1,8)*_ = 0.73 *p* = 0.42 *η*^*2*^_*p*_ = 0.08	*F*_*(1,8)*_ = 0.24 *p* = 0.64 *η*^*2*^_*p*_ = 0.03	***F***_***(1,8)***_** = 10.68** ***p* = 0.01** ***η***^***2***^_***p***_** = 0.57**	*F*_*(1,8)*_ = 0.74 *p* = 0.41 *η*^*2*^_*p*_ = 0.08	*F*_*(1,8)*_ = 0.18 *p* = 0.68 *η*^*2*^_*p*_ = 0.02	*F*_*(1,8)*_ = 4.41 *p* = 0.07 *η*^*2*^_*p*_ = 0.36	*F*_*(1,8)*_ = 0.22 *p* = 0.65 *η*^*2*^_*p*_ = 0.03
	*Right hand*	***F***_***(1,11)***_** = 5.36** ***p* = 0.04** ***η***^***2***^_***p***_** = 0.33**	***F***_***(1,11)***_** = 6.42** ***p* = 0.03** ***η***^***2***^_***p***_** = 0.37**			*F*_*(1,11)*_ = 0.30 *p* = 0.59 *η*^*2*^_*p*_ = 0.03	
	*Left hand*	*F*_*(1,8)*_ = 0.15 *p* = 0.71 *η*^*2*^_*p*_ = 0.02	***F***_***(1,8)***_** = 8.44** ***p* = 0.02** ***η***^***2***^_***p***_** = 0.51**			*F*_*(1,8)*_ = 1.86 *p* = 0.21 *η*^*2*^_*p*_ = 0.19	
TMDec%	*F*_*(1,8)*_ = 1.41 *p* = 0.27 *η*^*2*^_*p*_ = 0.15	*F*_*(1,8)*_ = 1.66 *p* = 0.23 *η*^*2*^_*p*_ = 0.17	***F***_***(1,8)***_** = 12.63** ***p* = 0.007** ***η***^***2***^_***p***_** = 0.61**	*F*_*(1,8)*_ = 2.94 *p* = 0.13 *η*^*2*^_*p*_ = 0.27	*F*_*(1,8)*_ = 0.64 *p* = 0.45 *η*^*2*^_*p*_ = 0.07	*F*_*(1,8)*_ = 0.001 *p* = 0.97 *η*^*2*^_*p*_ = 0.001	*F*_*(1,8)*_ = 0.93 *p* = 0.36 *η*^*2*^_*p*_ = 0.10
	*Right hand*	*F*_*(1,11)*_ = 2.23 *p* = 0.16 *η*^*2*^_*p*_ = 0.17	***F***_***(1,11)***_** = 27.77** ***p* < 0.001** ***η***^***2***^_***p***_** = 0.72**			*F*_*(1,11)*_ = 0.21 *p* = 0.66 *η*^*2*^_*p*_ = 0.02	
	*Left hand*	*F*_*(1,8)*_ = 2.69 *p* = 0.14 *η*^*2*^_*p*_ = 0.25	***F***_***(1,8)***_** = 5.35** ***p* = 0.05** ***η***^***2***^_***p***_** = 0.40**			*F*_*(1,8)*_ = 0.26 *p* = 0.63 *η*^*2*^_*p*_ = 0.03	

*Movement Time (MT).* The global ANOVA for MT revealed a significant main effect of Group. Post-hoc comparisons indicated that the DI group (M = 316.32 ms) had significantly longer MT than the DN group (M = 175.26 ms; mean difference = 141.06 ms, *p* = 0.003). A significant three-way interaction was found for Task × Hand × Group, indicating a complex pattern of group differences across tasks and hands. Bonferroni-corrected pairwise comparisons revealed that when browsing on Google with the Right hand, Digital Immigrants (M = 299.73; mean difference = 125.10 ms, p = 0.02) showed longer Movement Time than Digital Natives (M = 174.63 ms). This group difference for the Google task did not reach statistical significance when the participants used their left hand.

**Fig. 1:**
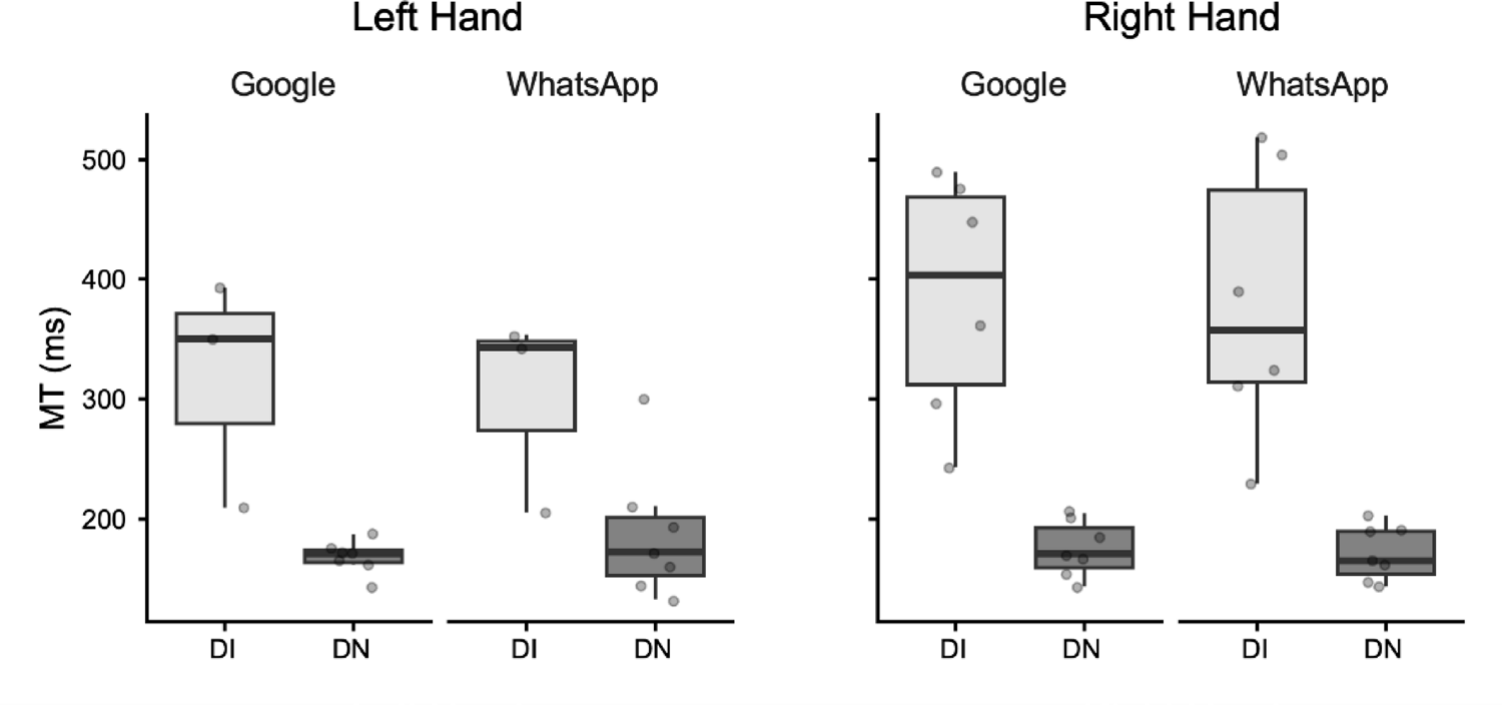
Box plots illustrating Movement Time (MT) in milliseconds (ms). Data are presented for Digitally Immigrant (DI, light grey boxes) and Digital Native (DN, dark grey boxes) groups. Results are separated by hand used (Left Hand, Right Hand) and application (Google, WhatsApp). The horizontal line within the box indicates the median, box boundaries represent the first and third quartiles, and whiskers extend to 1.5 times the interquartile range. Individual points represent data for single participants

Separate ANOVAs for MT by hand also revealed a significant main effect of Group for both the Right Hand (DI: M = 382.36 ms; DN: M = 172.93 ms; mean difference = 209.44 ms, *p* < 0.001) and the Left Hand (DI: M = 308.88 ms; DN: M = 177.60 ms; mean difference = 131.28 ms, *p* = 0.007) (Fig. 1).

*Maximum Velocity (MV).* The global ANOVA for MV revealed no significant main effect of Group. A significant main effect of Hand was observed. The right hand (M = 201.82 mm/s) exhibited higher MV values than the left hand (M = 134.37 mm/s; mean difference = 67.45, mm/s, *p* = 0.001).

**Fig. 2:**
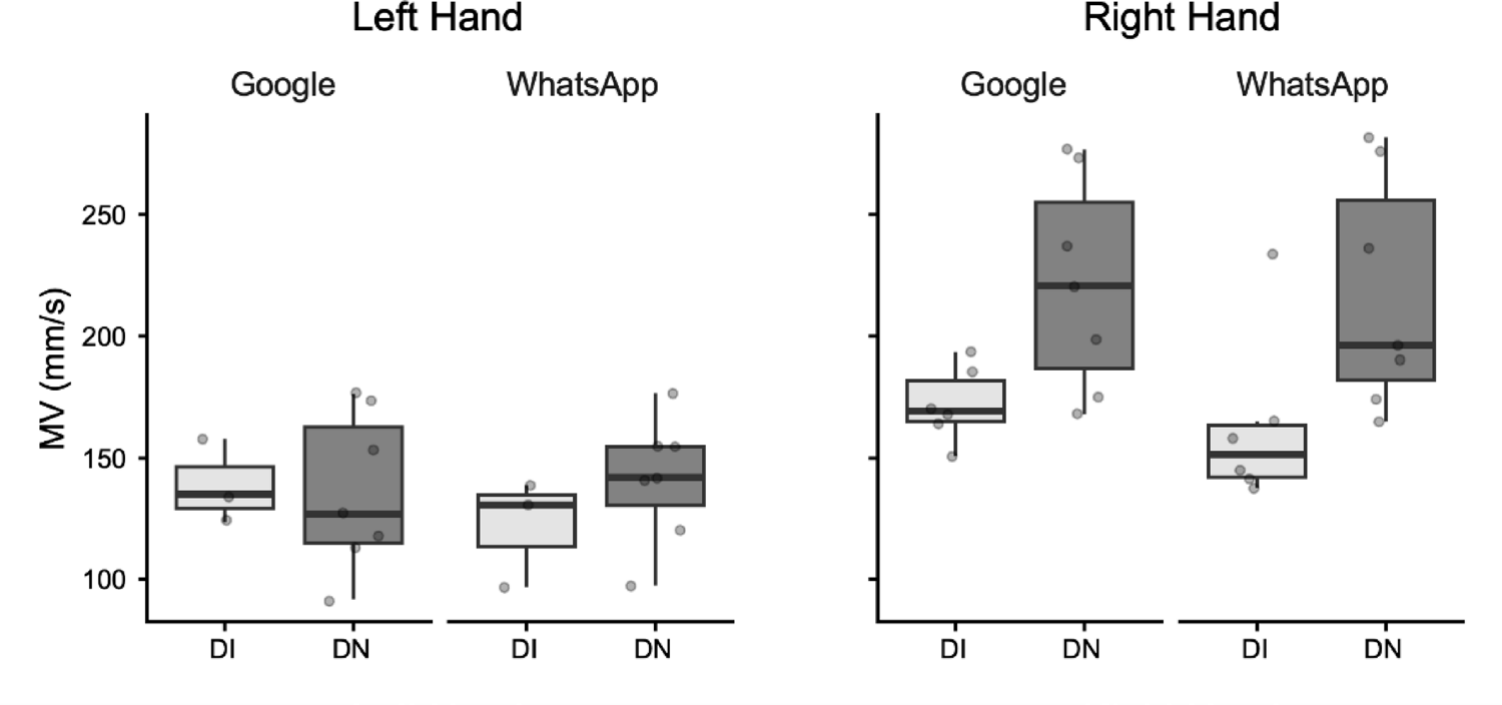
Box plots illustrating Maximum Velocity (MV) in millimeters per second (mm/s). Data are presented for Digitally Immigrant (DI, light grey boxes) and Digital Native (DN, dark grey boxes) groups. Results are separated by hand used (Left Hand, Right Hand) and application (Google, WhatsApp). The horizontal line within the box indicates the median, box boundaries represent the first and third quartiles, and whiskers extend to 1.5 times the interquartile range. Individual points represent data for single participants

For the Right Hand, a separate ANOVA showed a significant main effect of Group. The DI group (M = 167.74 mm/s) had significantly lower MV than the DN group (M = 219.21 mm/s; mean difference = − 51.47 mm/s, *p* = 0.03). For the Left Hand, a separate ANOVA revealed no significant main effect of Group (Fig. 2).

*Maximum Deceleration (MDec).* The global ANOVA for MDec revealed a significant main effect of Group. Post-hoc comparisons indicated that Digital Natives exhibited higher deceleration peaks (DI: M = 2141.65 mm/s^2^; DN: M = 3170.02 mm/s^2^; mean difference = − 1028.37 mm/s^2^, *p* = 0.04). A significant main effect of Hand was also found. The right hand (M = 3119.33 mm/s^2^) exhibited higher MDec values than the left hand (M = 2192.34 mm/s^2^; mean difference = 926.99 mm/s^2^, *p* = 0.01).

**Fig. 3:**
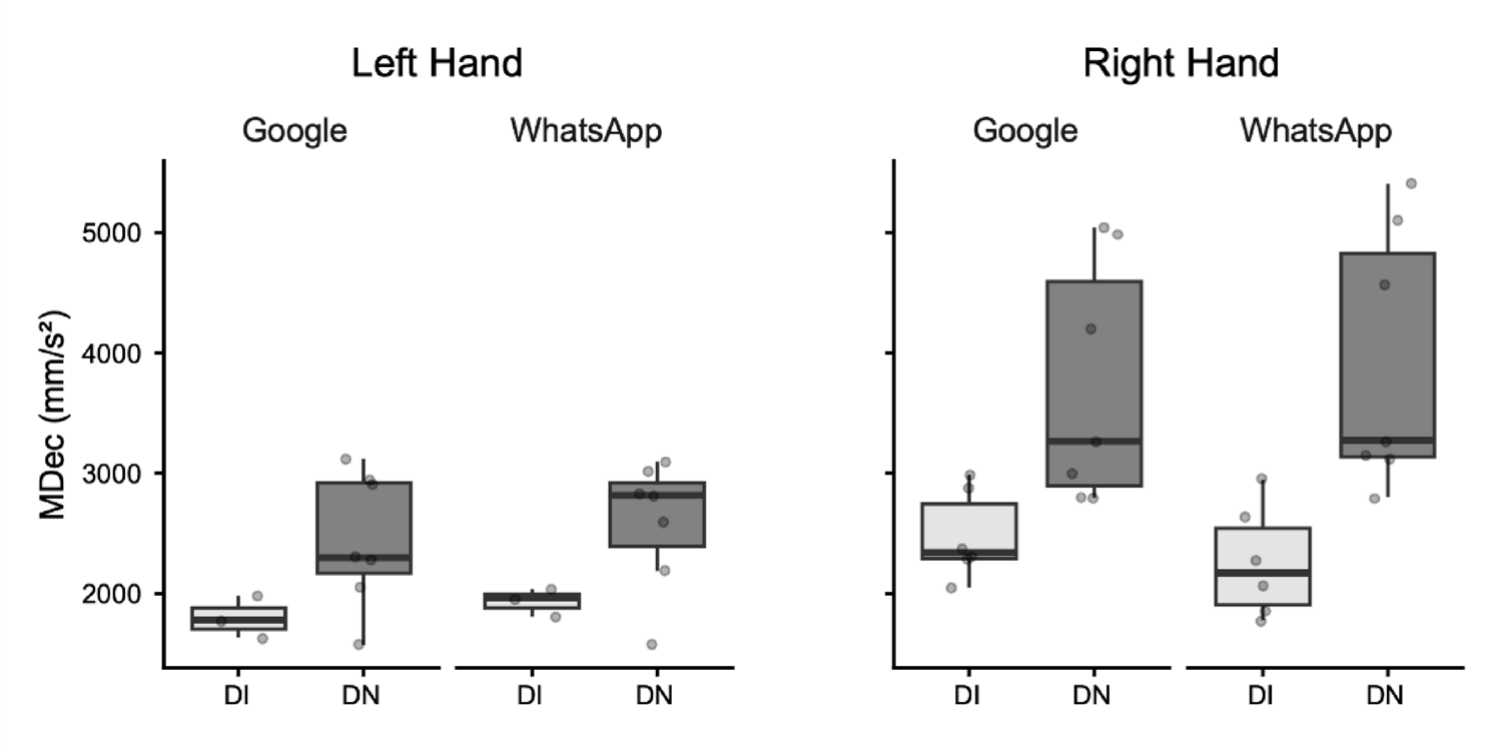
Box plots illustrating Maximum Deceleration (MDec) in millimeters per square second (mm/s^2^). Data are presented for Digitally Immigrant (DI, light grey boxes) and Digital Native (DN, dark grey boxes) groups. Results are separated by hand used (Left Hand, Right Hand) and application (Google, WhatsApp). The horizontal line within the box indicates the median, box boundaries represent the first and third quartiles, and whiskers extend to 1.5 times the interquartile range. Individual points represent data for single participants

For the Right Hand, a separate ANOVA revealed a significant main effect of Group (DI: M = 2366.46 mm/s^2^; DN: M = 3820.50 mm/s^2^; mean difference = − 1454.05 mm/s^2^, *p* = 0.007). For the Left Hand, a separate ANOVA indicated that the main effect of Group approached significance. The DI group (M = 1865.15 mm/s^2^) tended to have lower MDec than the DN group (M = 2519.54 mm/s^2^; mean difference = − 654.39 mm/s^2^, *p* = 0.06) (Fig. 3).

*Time to Maximum Velocity% (TMV%).* The global ANOVA for TMV% revealed a significant main effect of Group. The DI group (M = 38.58%) had significantly lower TMV% than the DN group (M = 47.01%; mean difference = − 8.43%, *p* = 0.01).

**Fig. 4:**
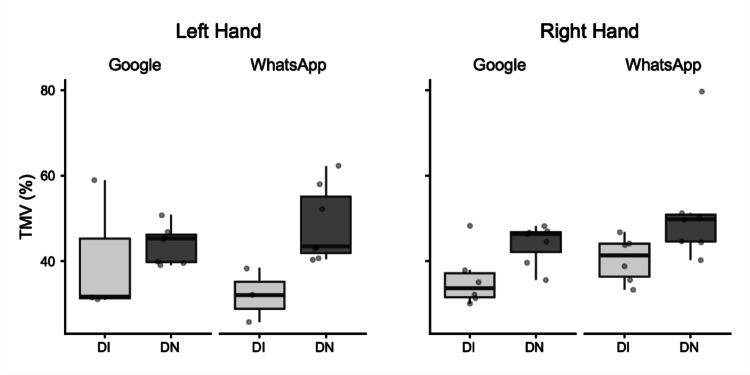
Box plots illustrating Time to Maximum Velocity (TMV) as a percentage (%). Data are presented for Digitally Immigrant (DI, light grey boxes) and Digital Native (DN, dark grey boxes) groups. Results are separated by hand used (Left Hand, Right Hand) and application (Google, WhatsApp). The horizontal line within the box indicates the median, box boundaries represent the first and third quartiles, and whiskers extend to 1.5 times the interquartile range. Individual points represent data for single participants

For the Right Hand, a separate ANOVA revealed a significant main effect of Group. The DI group (M = 38.13%) showed significantly lower TMV% than the DN group (M = 47.77%; mean difference = − 9.64%, *p* = 0.03). Crucially, for this hand, the main effect for Task was also significant, indicating that TMV% was higher for the WhatsApp task (M = 45.97%) compared to the Google task (M = 39.92%; mean difference = 6.05%, *p* = 0.04). For the Left Hand, a separate ANOVA also revealed a significant main effect of Group. The DI group (M = 36.29%) had significantly lower TMV% than the DN group (M = 46.24%; mean difference = − 9.95%, *p* = 0.02) (Fig. 4).

*Time to Maximum Deceleration% (TMDec%).* The global ANOVA for TMDec% revealed a significant main effect of Group. Post-hoc tests showed that DN, on average, showed significantly later peak deceleration in their movements (DI: M = 59.19%; DN: M = 79.64%; mean difference = − 20.45%, *p* = 0.007).

**Fig. 5:**
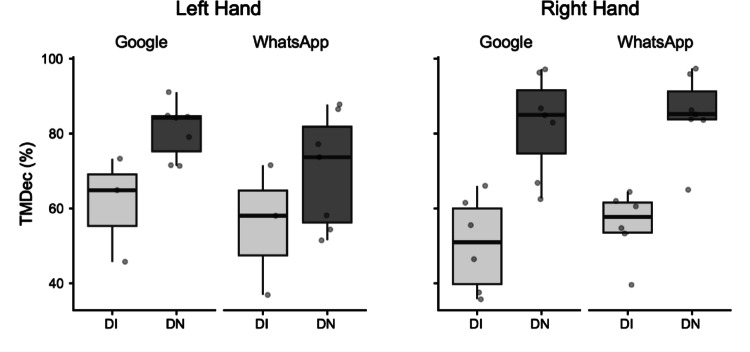
Box plots illustrating Time to Maximum Deceleration (TMDec) as a percentage (%). Data are presented for Digitally Immigrant (DI, light grey boxes) and Digital Native (DN, dark grey boxes) groups. Results are separated by hand used (Left Hand, Right Hand) and application (Google, WhatsApp). The horizontal line within the box indicates the median, box boundaries represent the first and third quartiles, and whiskers extend to 1.5 times the interquartile range. Individual points represent data for single participants

For the Right Hand, a separate ANOVA revealed a significant main effect of Group (DI: M = 53.07%; DN: M = 83.89%; mean difference = − 30.82%, *p* < 0.001). For the Left Hand, a separate ANOVA also revealed a significant main effect of Group (DI: M = 58.35%; DN: M = 75.39%; mean difference = − 17.04%, *p* = 0.05) (Fig. 5).

### Behavioral metrics (typing time, predictive text use, typing errors)

Video recordings capturing the participants' hands and the smartphone screen from above were analysed frame-by-frame to extract behavioural metrics and a detailed mapping of the spatial distribution of key presses across the virtual keyboard for both the right and left thumb.

Regarding the behavioural metrics, Typing Time was defined as the total time in seconds taken to type the entire sentence, computed by summing the interval between the first and last keystroke. Use of Predictive Text was defined as the frequency of predictive text selection events, specifically the number of times participants engaged with the on-screen predictive bar or Google’s auto-completion list, regardless of the number of preceding keystrokes or the orthographic accuracy of the typed string. This operationalization was adopted to reflect the participants’ motor strategy in naturalistic typing contexts. Typing Errors included all instances of typing errors and corrections, such as reversals, omissions, and additions. Outliers were identified and removed at the variable-specific level in line with standard practice for repeated-measures designs, thereby preserving the overall data integrity across measures. Data for the dependent variables were log-transformed to approximate normality prior to conducting the analyses.

For each metric, a mixed analysis of variance was conducted with Task (WhatsApp Chat, Google Search) as the within-subjects factor and Group (Digital Immigrants, Digital Natives) as the between-subjects factor.

**Fig. 6:**
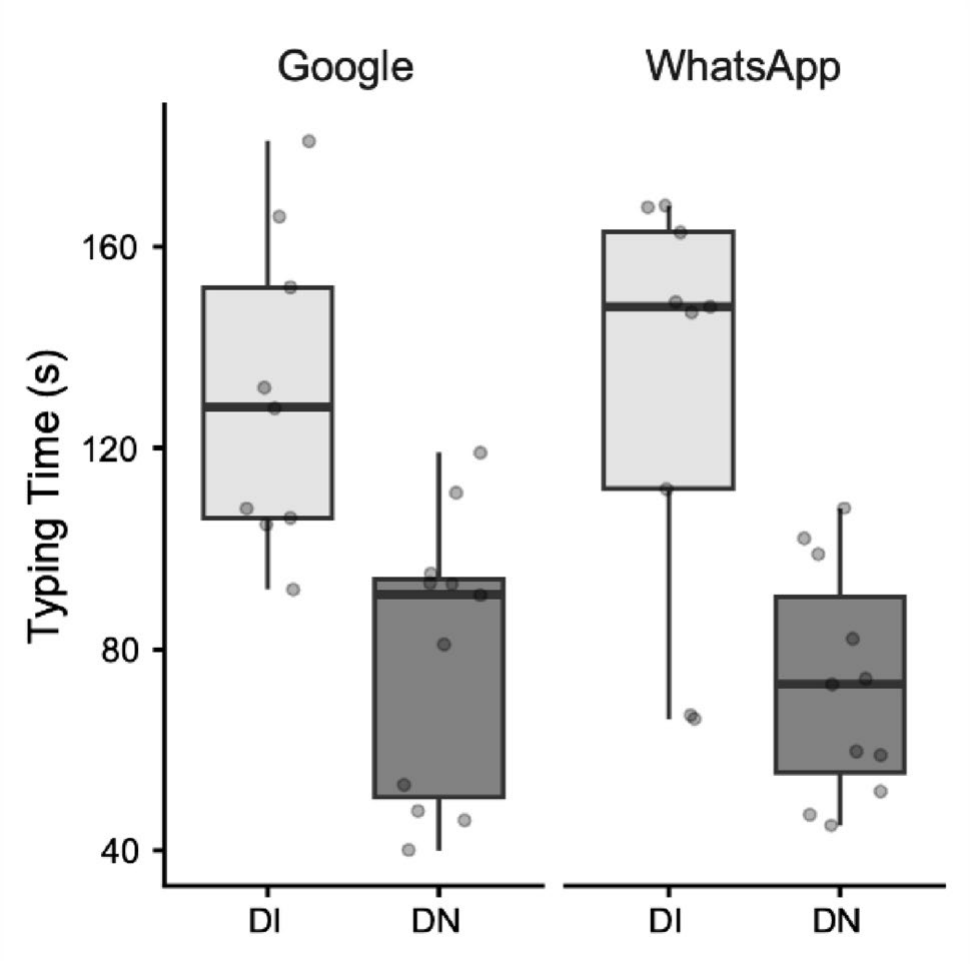
Box plots illustrating Typing Time in seconds (s). Data are presented for Digitally Immigrant (DI, light grey boxes) and Digital Native (DN, dark grey boxes) groups, across two applications: Google and WhatsApp. The horizontal line within the box indicates the median, box boundaries represent the first and third quartiles, and whiskers extend to 1.5 times the interquartile range. Individual points represent data for single participants

For Typing Time, the analysis revealed no significant main effect of Task (F_(1, 18)_ = 0.06, *p* = 0.08, *η*^*2*^ = 0.0007). However, there was a significant main effect of Group (F_(1, 18)_ = 26.15, *p* < 0.001, *η*^*2*^ = 0.47). Post-hoc comparisons indicated that the DI group (M = 130.99 s) had significantly longer typing time than the DN group (M = 75.97 s; mean difference = 55.03 s, *p* = 0.001). The Task x Group interaction was not significant (F_(1, 18)_ = 0.24, *p* = 0.63, *η*^*2*^ = 0.003) (Fig. 6).

**Fig. 7:**
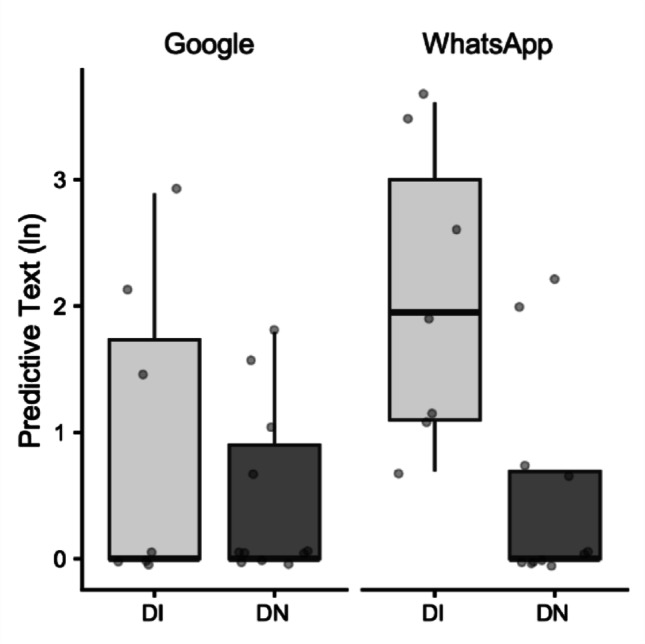
Box plots illustrating the mean number of predictive text usage. Data are presented for Digitally Immigrant (DI, light grey boxes) and Digital Native (DN, dark grey boxes) groups, across two applications: Google and WhatsApp. Data are log-transformed to correct for non-normality. The horizontal line within the box indicates the median, box boundaries represent the first and third quartiles, and whiskers extend to 1.5 times the interquartile range. Individual points represent data for single participants

Regarding the Use of Predictive Text, there was a significant main effect of Task (F_(1, 16)_ = 7.19, *p* = 0.016, *η*^*2*^ = 0.07). Post-hoc comparisons indicated that predictive text was used significantly more often in the WhatsApp task (M = 1.28) than in the Google task (M = 0.69; mean difference = 0.59, *p* = 0.02). A significant main effect of Group was also found (F_(1, 16)_ = 6.14, *p* = 0.03, *η*^*2*^ = 0.2), indicating that DI (M = 1.48) used predictive text more often than DN (M = 0.49; mean difference = 1, *p* = 0.03). Critically, there was a significant Task x Group interaction (F_(1, 16)_ = 6.46, *p* = 0.02, *η*^*2*^ = 0.06). This interaction suggests that the pattern of predictive text use across tasks differed between groups; specifically, DI showed a higher propensity to use predictive text in the WhatsApp Chat task (M = 2.06) compared to the Google Search task (M = 0.91, mean difference = 1.66, *p* = 0.02). This task difference for predictive text use did not reach statistical significance in DN group (Fig. 7).

For Typing Errors, the analysis showed no significant main effect of Task (F_(1, 15)_ = 0.19, *p* = 0.66, *η*^*2*^ = 0.002). There was also no significant main effect of Group (F_(1, 15)_ = 0.46, *p* = 0.51, *η*^*2*^ = 0.02). The Task x Group interaction was not significant (F_(1, 15)_ = 0.20, *p* = 0.66, *η*^*2*^ = 0.002). This indicates that the number of typing errors and corrections did not significantly differ between tasks or between the two groups.

### Thumb usage patterns

To quantify how participants utilized their left and right thumbs across the keyboard, we performed a detailed video analysis of each key press, adapting the approach by Yu et al. (2023). For each letter key and the spacebar, we recorded whether it was pressed by the left or right thumb. To ensure comparability across conditions, we only included key presses for letters explicitly typed in both tasks (excluding letters completed by predictive text in one but not the other), for each sentence and participant. We computed the Thumb Usage Rate (TU) for each thumb (Right: TUR; Left: TUL), defined as the percentage of total touches on a given key performed by that thumb. Given the non-normal distribution of TU data, non-parametric tests were used.

**Fig. 8:**
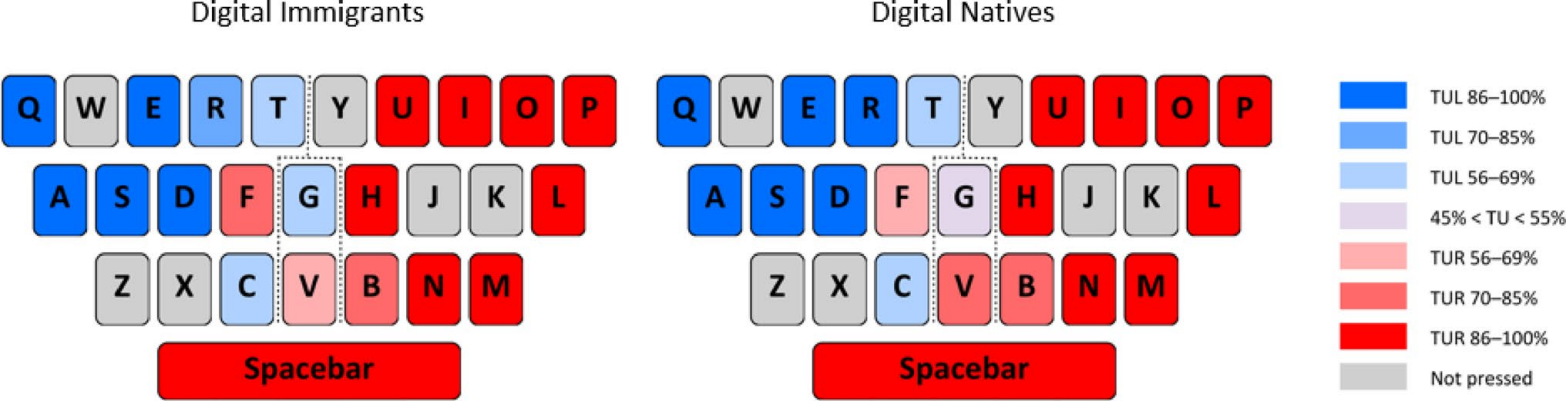
Schematic representation of Right Thumb Usage Rate (TUR) and Left Thumb Usage Rate (TUL) for all letter keys and the spacebar, shown for Digital Immigrant (on the left) and Digital Native (on the right) groups. Thumb Usage Rate represents the proportion of total key presses performed by one thumb on a given key. The keyboard is delineated into left, middle, and right sections by a dotted line. The color legend on the right indicates the percentage of left (blue shades) or right (red shades) thumb usage rate for each key, as well as keys with mixed usage or those not pressed

Binomial tests (*α* = 0.05) were first conducted on the overall sample to identify keys predominantly used (> 50%) by one thumb, and those showing a strong preference (> 85%). These tests confirmed a general right thumb dominance, with 12 keys (including the Spacebar) predominantly pressed by the right thumb and 8 by the left (> 50%). For strong preference (> 85%), the right thumb dominated 9 keys, and the left 5. Separate binomial tests for the DI and DN groups revealed largely similar patterns of thumb-to-key mapping, indicating that both cohorts primarily used the right thumb for keys on the right side and the left thumb for keys on the left, with central keys showing more variability. Specifically, in the DI group, the right thumb was predominant for 12 keys and the left for 7 (> 50%), with 9 keys showing strong right thumb preference and 5 strong left thumb preference (> 85%). In the DN group, the right thumb was predominant for 11 keys and the left for 8 (> 50%), with 8 keys showing strong right thumb preference and 6 strong left thumb preference (> 85%) (Fig. 8).

Subsequently, according to Yu et al. (2023), we examined potential differences in thumb usage rates (TUR/TUL) between tasks and groups for a subset of keys known to show mixed usage (those with TUR/TUL below 85% overall), plus V and G keys which were included due to their central position on the keyboard. The final subset of examined keys was B, C, F, G, R, T, and V. Within-group Wilcoxon signed-rank tests found no significant differences in TUR or TUL values between the WhatsApp and Google conditions for the selected keys in either the DI or DN group. Between-group Mann–Whitney U tests revealed no significant differences in thumb usage between the DI and DN groups.

## Discussion

This study aimed to unravel how distinct digital experience histories and task intentions shape the kinematic profiles and behavioral strategies employed during touchscreen typing. By comparing individuals from the early digital exposure group (DN) and the later digital adoption group (DI) across WhatsApp Chat (content generation) and Google Search (content consumption) tasks, our findings provide compelling evidence that lifelong sensorimotor interaction with digital devices fundamentally reconfigures fine-grained motor skills and adaptive strategies.

A primary and consistent finding across multiple kinematic metrics is the superior motor efficiency and "motor vigor" (Summerside et al. 2018; Shadmehr et al. 2019) – the speed and intensity with which an action is executed – exhibited by the group with early digital exposure (DN) compared to the group with later digital adoption (DI). DN consistently displayed shorter Movement Times (MT), indicating faster execution of individual thumb movements between key presses. This kinematic advantage was coupled with a higher-vigor motor profile, characterized by significantly higher Maximum Deceleration (MDec) across both hands, and higher Maximum Velocity (MV) specifically for the dominant right hand. Furthermore, the timing of peak velocity (TMV%) and peak deceleration (TMDec%) within the movement cycle differed markedly between groups: DN reached their peak velocity and deceleration later in the movement, suggesting a more ballistic, pre-planned, and highly controlled movement trajectory (Zelaznik et al. 1986). This pattern, where rapid acceleration is followed by a sharp, late deceleration, is characteristic of highly skilled and efficient motor performance, allowing for rapid approach to the target with precise braking (Djioua and Plamondon 2010). In contrast, DI's earlier peak velocity and deceleration might indicate a more segmented, cautious, or less fluid movement control strategy, potentially requiring more online adjustments (Buchanan 2013). These kinematic distinctions align perfectly with principles of motor learning, where extensive and early exposure to a specific motor skill leads to increased automaticity, reduced movement variability, and enhanced efficiency (Davids et al. 2003). For DN, their pervasive interaction with touchscreens from an early age has likely fostered the development of highly optimized sensorimotor programs for digital input, akin to expertise in a physical skill. While it is well-documented that motor vigor and processing speed tend to decline with physiological aging (Shadmehr et al. 2010; De Riggi et al. 2025), suggesting that age likely contributes to the observed slowness in the DI group, the specific kinematic patterns observed here (ballistic vs. controlled) closely resemble differences observed between experts and novices in other motor domains (Sosnik et al. 2004; Elliott et al. 2010). This suggests that digital experience is a key driver beyond simple biological aging. Moreover, considering that our Digital Immigrant group (mean age 58) corresponds demographically to the healthy 'younger' baseline in recent aging studies (De Riggi et al. 2025), it is unlikely that the observed kinematic differences are driven solely by advanced motor decline, reinforcing the interpretation of specific sensorimotor history effects.

Behaviorally, the kinematic superiority of DN translated directly into overall faster typing times compared to DI, reinforcing their enhanced motor efficiency. Crucially, despite these significant differences in speed and movement quality, both groups maintained comparable levels of typing accuracy, as evidenced by the lack of significant differences in typing errors. This suggests that while DI may be kinematically less efficient, they effectively compensate to maintain performance output.

The observed compensatory mechanisms and strategic flexibility of Digital Immigrants are further illuminated by their patterns of predictive text use. DI leveraged predictive text significantly more often than DN, particularly in the WhatsApp Chat task. This finding is highly insightful. The WhatsApp Chat task, representing content generation and communication, aligns with the concept of "near" digital space (Craighero & Marini 2021), potentially carrying a perceived communicative pressure. For DI, who are kinematically less efficient, relying on predictive text in this context serves as a strategic cognitive offloading mechanism, allowing them to maintain conversational flow and accuracy without solely depending on high-vigor, fine-motor execution. In contrast, DN, with their superior motor control, did not show a significant modulation of predictive text use across tasks, suggesting their motor fluency is robust enough not to necessitate such strategic aids or that they are simply less inclined to use them habitually. The complex three-way interaction for Movement Time (Task x Hand x Group) further elucidates the nuanced, context-dependent demands placed on DI's motor control, revealing situations where their kinematic efficiency is notably challenged. Specifically, DI exhibited a significantly longer Movement Time with their right hand when performing the Google Search task compared to DN. A possible explanation for this finding is that the Google search task, focused on content consumption and information retrieval, may impose a higher cognitive load on DI compared to the WhatsApp chat. This elevated cognitive demand could stem from the need for precise query formulation, the continuous evaluation of suggested options (if available and perceived as helpful), and the planning of subsequent navigation. If DI allocate a greater proportion of their cognitive resources to these higher-level processes, fewer resources might remain available for fluid and rapid motor execution, leading to the observed increase in Movement Time (i.e., a cognitive-motor interference phenomenon). Conversely, DN do not exhibit this effect, likely owing to their superior motor control and highly automatized digital skills. The WhatsApp task, being a conversational activity, might be perceived by DI as a more familiar or socially guided task, where fluidity of thought and typing is prioritized. While the full implications of this specific interaction warrant further investigation, it hints at subtle, context-dependent adaptations in motor efficiency, potentially reflecting distinct cognitive representations of digital space and task goals.

The analysis of thumb usage patterns revealed no major differences between groups or tasks in terms of which thumb was used for specific keys, despite an overall right thumb dominance. This suggests that the mapping of keys to thumbs on a virtual keyboard is a largely conserved motor strategy across generations, likely influenced by the standardized layout of the virtual keyboard itself rather than differential sensorimotor histories. This finding indicates that while *how* movements are performed kinematically differs, the fundamental *what* (which thumb for which key) is broadly consistent.

Collectively, these findings support the embodied cognition framework (Wilson 2002; Gallese and Lakoff 2005; Anderson et al. 2012) by demonstrating that an individual's sensorimotor history is a key factor shaping their interaction with the digital world. The group with early digital exposure (Digital Natives), having grown up within the digital ecosystem, exhibit motor control characteristics indicative of highly practiced, efficient, and vigorous execution, akin to native speakers of a language. Their digital fluency appears rooted in deeply ingrained sensorimotor programs. In contrast, the group with later digital adoption (Digital Immigrants), while less kinematically "native," are not simply less capable; rather, they demonstrate remarkable strategic flexibility. They adapt their motor control (e.g., modulating deceleration, though less pronounced than DN) and leverage available interface features (e.g., predictive text) to achieve task goals effectively. This highlights that "digital fluency" is a multifaceted construct, encompassing not only raw motor speed and efficiency but also sophisticated adaptive strategies to navigate the demands of diverse digital environments. The differences observed are not merely a matter of age or general tech-savviness, but rather reflect distinct sensorimotor "dialects" developed through divergent interactional histories with the physical and digital worlds. Thus, while both groups possess the 'habit' of daily smartphone use, the 'sensorimotor history' of early adoption appears to confer a distinct advantage in motor automaticity that late adulthood practice cannot fully replicate.

Despite the robust findings, this study has several limitations. The sample size for the detailed kinematic analysis of consecutive key presses, though carefully selected for data quality, was relatively small. A crucial aspect related to the study's design is the inherent variability introduced by conducting the experiment under ecological conditions to capture naturalistic smartphone interaction. While this approach enhances external validity, it necessarily leads to considerable variability in movement data, especially for the analysis of consecutive key presses. Figures 1–5 vividly illustrate this variability, particularly notable in the wider distributions and scattered individual data points for the Digital Immigrant group, and also reflect the uneven distribution of comparable consecutive movements between hands and groups as detailed in Table 1. Notably, the reduced sample size for the Left Hand in the DI group reflects the naturalistic difficulty older adults faced in performing consecutive keystrokes with the non-dominant thumb, leading to data exclusion based on our strict quality criteria. Indeed, achieving a perfectly balanced number of consecutive key presses across all participants and conditions is a significant methodological challenge in naturalistic typing studies. While a greater number of trials could potentially allow for more balanced data sets through selective inclusion, this would come at the cost of significantly increased participant burden and experimental duration, potentially impacting task naturalness or leading to fatigue. This inherent variability, while acknowledged and managed through careful data selection and statistical methods (e.g., exclusion of outliers based on deciles), represents a conscious trade-off for capturing authentic, unconstrained typing behavior.

Furthermore, while our findings support the notion that divergent digital history contributes to shaping sensorimotor skills and adaptive strategies in touchscreen typing, it is crucial to acknowledge a significant confounding factor: chronological age. Our Digital Immigrant group is, by definition, inherently older than the Digital Native group. Research, such as that by Ceolini et al. (Ceolini et al. 2022), highlights that smartphone interaction patterns can serve as quantifiable biomarkers for healthy and pathological aging. This suggests that some of the observed differences in motor performance and interaction patterns could, in part, be attributed to age-related changes in motor control, cognitive processing speed, or general physical dexterity, rather than solely to a different history of digital interaction. Given the inherent correlation between age and digital adoption trajectories in current generational cohorts, our study, like many others comparing Digital Immigrants and Digital Natives, cannot definitively disentangle the independent contributions of chronological age versus the specific digital experience. Future research employing longitudinal designs or examining age-related differences within a homogenous group of Digital Natives (i.e., individuals with similar early digital exposure but varying chronological ages) would be instrumental in isolating these effects and providing a clearer understanding of the distinct roles of age and digital experience.

Moreover, the use of participants' own smartphones, while enhancing ecological validity, introduced variability in device characteristics (e.g., screen size, keyboard layout, predictive text algorithms) that could subtly influence typing kinematics and behavioral patterns across individuals. While our kinematic analysis focused on relative movements between keys, absolute key positions varied. Future research could control for device characteristics or use a standardized testing environment. Additionally, our study focused on two specific digital intentions (chat and search) using predefined sentences; future research could explore a broader range of common smartphone interactions such as complex multi-page navigation (e.g., as encountered when copying an OTP for a bank transfer) or conversational AI interactions, which uniquely challenge the distinction between human-to-human communication (as in Whatsapp chat) and querying a non-social information source (as in Google search). Moreover, exploring a broader range of digital interactions, such as swiping or scrolling, could reveal further nuanced kinematic and behavioral differences. Lastly, while our study provides objective behavioral and kinematic signatures, future studies could investigate the neural correlates of these generational differences in sensorimotor control, potentially identifying distinct brain network activations or structural adaptations underlying "digital nativeness."

## Conclusion

This study provides compelling evidence that an individual's sensorimotor history contributes to shaping fine-grained sensorimotor skills and adaptive strategies during touchscreen typing. Individuals with early and pervasive digital exposure consistently demonstrate superior motor efficiency, characterized by faster, more vigorous, and kinematically optimized thumb movements, reflecting a deeply ingrained sensorimotor fluency acquired through extensive early exposure to digital interfaces.

In contrast, individuals with later digital adoption, while exhibiting lower kinematic efficiency, maintain performance accuracy by employing sophisticated adaptive strategies. Their strategic reliance on interface features like predictive text, particularly in communication-focused tasks, underscores their ability to compensate for kinematic differences and tailor their approach to task demands.

These findings highlight that "digital fluency" is not merely a monolithic skill but a complex interplay of motor efficiency and strategic flexibility, shaped by an individual's unique sensorimotor developmental trajectory within the evolving digital landscape. Understanding these divergent sensorimotor histories and the resulting adaptive strategies is crucial for designing more intuitive digital interfaces, developing targeted digital literacy programs, and appreciating the diverse pathways through which individuals navigate the demands of the digital age.
